# Transcriptomic Comparisons of Somatic and Cancer Stem Cells

**DOI:** 10.3390/biomedicines13082039

**Published:** 2025-08-21

**Authors:** Austin Drysch, Arun Ahuja, Dillan Prasad, Rishi Jain, Sharbel Romanos, Amr Alwakeal, Christopher Ahuja

**Affiliations:** Department of Neurological Surgery, Northwestern University Feinberg School of Medicine, Chicago, IL 60611, USA; arun.ahuja@northwestern.edu (A.A.); dillan.prasad@northwestern.edu (D.P.); rishi.jain@northwestern.edu (R.J.); sharbel.romanos@northwestern.edu (S.R.); amr.alwakeal@northwestern.edu (A.A.); christopher.ahuja@northwestern.edu (C.A.)

**Keywords:** cancer stem cells, somatic stem cells, transcriptomics, stemness, tumor microenvironment, signaling pathways, precision medicine, regenerative medicine

## Abstract

Stem cells are essential for tissue maintenance, repair, and regeneration, yet their dysregulation gives rise to cancer stem cells (CSCs), which drive tumor progression, metastasis, and therapy resistance. Despite extensive research on stemness and oncogenesis, a critical gap remains in our understanding of how the transcriptomic landscapes of normal somatic stem cells (SSCs) diverge from those of CSCs to enable malignancy. This review synthesizes current knowledge of the key signaling pathways (Wnt, Notch, Hedgehog, TGF-β), transcription factors (Oct4, Sox2, Nanog, c-Myc, YAP/TAZ), and epigenetic mechanisms (chromatin remodeling, DNA methylation, microRNA regulation) that govern stemness in SSCs and are hijacked or dysregulated in CSCs. We highlight how context-specific modulation of these pathways distinguishes physiological regeneration from tumorigenesis. Importantly, we discuss the role of epithelial–mesenchymal transition (EMT), cellular plasticity, and microenvironmental cues in reprogramming and maintaining CSC phenotypes. By integrating transcriptomic and epigenetic insights across cancer biology and regenerative medicine, this review provides a framework for identifying vulnerabilities specific to CSCs while still preserving normal stem cell function. Understanding these distinctions is essential for the development of targeted therapies that minimize damage to healthy tissues and advance precision oncology.

## 1. Introduction

The ability of cancer stem cells (CSCs) to self-replenish and heterogeneously differentiate significantly influences disease severity [1]. Recently, consensus has emerged supporting the model of cancer, which sees CSCs as a critical driver of metastatic potential, progression, and therapeutic resistance. Consequently, CSCs are a primary therapeutic target for aggressive diseases [2].

CSCs and somatic stem cells (SSCs) share common hallmark features that are thought to contribute to stemness and plasticity [3]. While many phenotypic and morphologic similarities have been reported between the two cell types, it is the differences between CSCs and SSCs that may offer critical therapeutic relevance. Discovering—and targeting—the unique signaling pathways of CSCs may enable researchers to deliver better treatments and, ultimately, better outcomes for patients.

## 2. Understanding Regenerative Cell Stemness

Regenerative medicine leverages the remarkable ability of stem cells to differentiate into diverse cell types, offering a transformative approach to chronic disease treatment. SSCs play a particularly vital role in tissue repair and inflammation modulation, holding great promise for diseases that are often unresponsive to traditional medicines [4]. To date, therapeutic stem cell research has primarily focused on developing treatments to prevent inflammation, with mesenchymal stem cells (MSCs) standing out for their ability to modulate immune responses and promote tissue regeneration [5]. In inflammatory bowel disorders, MSCs help restore the intestinal barrier and repair damaged mucosa [6,7]. Similarly, in rheumatoid arthritis, MSCs alleviate inflammation and enhance joint function, thereby mitigating symptoms and slowing disease progression [8]. MSC-based therapies are also under investigation for sepsis, where they have shown potential in reducing systemic inflammation and improving survival rates among critically ill patients [9]. These findings underscore the wide-ranging therapeutic capabilities of MSCs in managing inflammatory disorders.

Regenerative stem cells possess several key characteristics collectively described as “stemness”, particularly their ability to self-renew and differentiate into multiple downstream cell types. Transcriptomic studies have been pivotal in elucidating the regulatory networks that maintain the undifferentiated state or drive differentiation into specific progeny [10]. These mechanisms involve complex signaling pathways and genetic factors that govern the balance between maintaining stemness and initiating differentiation. This balance is critical for normal development and for the maintenance of tissue homeostasis in adults [10]. A major breakthrough in understanding stemness was Takahashi and Yamanaka’s discovery that specific factors can induce pluripotency in somatic cells, creating the field of induced pluripotent stem cells (iPSCs) [4]. These iPSCs, capable of differentiating along all three germ cell layers, serve as invaluable tools for studying development and disease while offering significant therapeutic potential.

Ongoing clinical trials utilizing iPSCs are advancing regenerative medicine in areas such as Parkinson’s disease, cardiac regeneration, and retinal regeneration. For Parkinson’s disease, trials are investigating both autologous and allogeneic iPSC-derived dopaminergic neurons. Autologous approaches, such as those led by Aspen Neuroscience, involve reprogramming patients’ own cells into dopaminergic neurons to reduce immune rejection, while companies like BlueRock Therapeutics focus on allogeneic products for scalable therapies [11,12,13,14,15]. In cardiac regeneration, clinical trials are evaluating allogeneic iPSC-derived cardiomyocytes for repairing damaged heart tissue, building on animal studies that demonstrated their integration into the host myocardium and improvement of heart function [16,17,18]. For retinal regeneration, iPSC-derived retinal pigment epithelium (RPE) cells are under investigation for treating age-related macular degeneration (AMD) [19]. Since 2017, clinical studies in Japan have shown promising results with autologous RPE cells transplanted into AMD patients, improving retinal structure and function [20]. These trials thus demonstrate the potential of iPSC-based therapies in addressing degenerative diseases.

Transcriptomics plays a pivotal role in understanding the biological foundations of stemness by identifying salient genes and pathways. RNA sequencing (RNA-seq) has been instrumental in elucidating gene expression dynamics during differentiation, revealing how intrinsic, extrinsic, and microenvironmental factors collectively shape transcriptomic profiles to achieve specific therapeutic outcomes in stem cell research [10,21]. For instance, single-cell RNA sequencing (scRNA-seq) has identified WNT4 and WNT2B signaling as key drivers of unwanted differentiation into neural and melanocyte lineages during chondrogenesis, respectively. By specifically targeting intrinsic pathways such as WNT and MITF for inhibition, researchers have successfully improved both the homogeneity and yield of cartilage-forming cells [22]. Similarly, whole transcriptomic analysis has provided insights into how extrinsic factors like cytokines, such as IL-1β, shape the inflammatory microenvironment and influence cellular behavior. RNA-seq studies revealed that IL-1β exposure alters fibroblast gene expression, upregulating CXCL chemokines (e.g., CXCL1, CXCL8) that hinder epithelial progenitor growth. Blocking this signaling pathway restores fibroblast support, underscoring the importance of balancing inflammation for effective tissue repair [23].

Additionally, RNA-seq research has demonstrated the role of DNMT3A, a DNA methyltransferase, in regulating alternative splicing events that act as molecular switches, controlling the transition from a stem cell state to specific lineage commitments [24]. Whole transcriptomic analyses of MSCs cultured in engineered micro-scaffolds have further revealed how the physical environments influence gene expression. Notably, 3D scaffolds promote the expression of genes associated with stemness, including those involved in the actin cytoskeleton, extracellular matrix, and cell adhesion molecules [25]. These findings highlight the profound impact of the physical microenvironment on stem cell behavior, offering promising strategies to modulate stemness and differentiation for therapeutic applications.

## 3. Overview of Cancer Stem Cells

First identified in leukemia in 1994, CSCs are increasingly recognized as crucial players in the initiation, progression, and resistance of tumors. CSC functionality includes self-renewal, tumor initiation, and adaptation to environmental cues, features that complicate treatment with conventional therapies [2,26]. Despite constituting a small fraction of tumor mass, CSCs are capable of regenerating tumors independently, rendering them key drivers of metastasis and recurrence [27,28]. CSCs are partially defined by specific cell surface markers, including CD133, CD44, CD90, EpCAM, and ALDH; however, the variability of these markers across tumor types poses challenges for their use as universal therapeutic targets [26,28,29]. Nevertheless, these markers facilitate the identification and isolation of CSCs from heterogeneous tumor populations and enable the development of precision therapies targeting CSC-specific survival mechanisms [28,30].

The self-renewal and plasticity of CSCs enable them to sustain tumorigenesis and respond dynamically to environmental cues, a process often regulated by the tumor microenvironment and key transcription factors [28]. The CSC niche, composed of elements such as the extracellular matrix, tumor-associated macrophages (TAMs), and cancer-associated fibroblasts (CAFs), plays a pivotal role in supporting CSC plasticity and survival under therapeutic pressure [30,31]. Hypoxia, a common physiological condition within tumors, further activates hypoxia-inducible factors (HIFs), which regulate key signaling pathways including Wnt/β-catenin, TGF-β, and NF-κB [28]. Collectively, the CSC niche sustains stemness, promotes CSC survival, and enhances resistance to therapies, complicating efforts to eradicate these cells [28,30,32]. The CSC niche additionally provides signals that maintain CSCs in their undifferentiated state, making the microenvironment a critical target for disrupting CSC function and overcoming resistance to therapy [31].

The origins of CSCs are multifactorial and involve the transformation of SSCs or early progenitor cells under the influence of both intrinsic and extrinsic factors. SSCs reside in well-characterized anatomical niches that support tissue maintenance and regeneration. These include hematopoietic stem cells within the bone marrow, neural stem cells in the subventricular zone of the brain, intestinal crypt stem cells, and epidermal basal layer stem cells. Additional niches include the bulge region of hair follicles, skeletal muscle satellite cells, and liver progenitor cells. These environments are finely tuned to preserve stem cell quiescence, self-renewal, and differentiation capacity [33,34,35].

In vivo, SSCs maintain a precise balance between self-renewal and differentiation through the integration of spatial cues, dynamic transcriptional regulation, and intrinsic polarity mechanisms. In the intestinal crypt, for example, a short-range Wnt gradient secreted by stromal niche cells such as telocytes and pericryptal myofibroblasts preserves stem cell identity at the crypt base, while graded BMP signaling promotes differentiation toward the villus [36,37]. This spatial organization is reinforced by oscillatory expression of transcription factors like Sox2 and c-Myc, which mediate transitions between quiescence, proliferation, and lineage commitment [38,39]. Additionally, asymmetric cell division, guided by Wnt-mediated mitotic spindle orientation and polarity complexes such as PAR proteins ensures that one daughter cell retains stemness while the other differentiates [40,41,42]. Together, these extrinsic and intrinsic mechanisms enable SSCs to regenerate tissue without uncontrolled expansion or loss of identity.

Transformation into CSCs can be driven by several triggers. Chronic inflammation promotes oxidative stress and aberrant cytokine signaling, accelerating genomic instability and stem cell exhaustion [43,44]. Hypoxia induces reprogramming through activation of HIFs and reshapes metabolic and epigenetic profiles that favor stem-like behavior [45]. Genetic alterations such as oncogene activation (e.g., KRAS, Myc) or tumor suppressor loss (e.g., TP53, APC) directly disrupt the regulatory mechanisms governing cell cycle and differentiation [46]. Therapy-induced plasticity is another important mechanism, wherein cytotoxic or targeted therapies create selective pressure that favors CSC survival or reprogram non-stem cancer cells into a CSC-like phenotype [47]. Among these, chronic inflammation and hypoxia are especially important not only in triggering CSC emergence but also in sustaining CSC populations via ongoing cytokine signaling, epigenetic modulation, and metabolic adaptation [43]. Understanding these converging mechanisms provides a basis for therapeutic strategies that aim to prevent CSC formation or selectively eliminate existing CSCs while sparing normal stem cells. The transcriptional landscape of CSCs is governed by a network of transcription factors that regulate stemness, differentiation, and resistance. Key among these are Oct4, Sox2, c-Myc, KLF4, Nanog, and YAP/TAZ—the same factors that play a role in the generation of iPSCs, where their overexpression reprograms somatic cells to a pluripotent state and enables cellular plasticity [26]. These factors also govern the transition of CSCs across phenotypic states in response to environmental cues, facilitating CSC survival under therapeutic pressure [26,28]. This intricate regulatory network underscores the complexity of targeting CSCs and highlights the need for innovative strategies to address their role in tumor progression and therapy resistance.

## 4. Cell Surface Signaling Regulators in SSCs and CSCs

Cell surface molecules play critical roles in the regulation of stemness, self-renewal, differentiation, and interaction with the microenvironment in both SSCs and CSCs (Table 1). These molecules not only serve as useful markers for the identification and isolation of stem-like cell populations but also function as key mediators of intracellular signaling cascades. They regulate pathways such as Wnt/β-catenin, Notch, and PI3K/AKT, which control cell fate decisions, immune evasion, chemoresistance, and metastatic potential [27,28].

As previously discussed, surface markers such as CD133 (prominin-1), CD44, and EpCAM are widely used for enrichment and functional studies in CSCs. CD133 is implicated in tumorigenesis, therapeutic resistance, and activation of oncogenic signaling pathways. It enhances CSC survival by promoting Wnt/β-catenin and PI3K/AKT signaling [48,49]. CD44, a hyaluronan receptor, regulates cell adhesion, migration, and EMT and is associated with increased tumor aggressiveness and poor prognosis [50,51]. EpCAM plays dual roles in mediating cell–cell adhesion and modulating EMT and proliferation through Wnt signaling. Its clinical relevance is made clear by the approval of anti-EpCAM antibody–drug conjugates, such as catumaxomab, in Europe for the treatment of malignant ascites [52,53].

Other important CSC-associated surface molecules include CD24, LGR5, ICAM1 (CD54), and ALDH1. CD24 modulates tumorigenic pathways such as src/FAK and GLI1 and is linked to increased metastatic potential [54]. LGR5, a known Wnt target gene, marks adult stem cells and CSCs with high tumor-initiating capacity and chemotherapy resistance [55]. ICAM1 facilitates immune evasion and metastasis, while ALDH1, which is involved in detoxification, serves as both a functional enzyme and stemness marker that contributes to chemoresistance [56,57].

Conversely, in SSCs, classical markers include CD34, integrins (e.g., α6, β1), and Notch receptors. CD34 is a well-characterized marker of hematopoietic and endothelial progenitors and plays a role in cell adhesion and maintenance of an undifferentiated state [58]. Integrins mediate cell–extracellular matrix (ECM) adhesion and mechanotransduction, regulating stemness, quiescence, and directional migration in both SSCs and CSCs [59]. Notch receptors are essential for maintaining stem cell compartments by promoting self-renewal and preventing premature differentiation, and they play similar roles in CSCs [60].

Despite these differences, the heterogeneity of tumor-associated markers across cancer types and the overlap in surface markers between somatic and cancer stem cells have thus far limited the development of effective targeted therapies. As a result, precision oncology approaches will likely require the identification of more specific or context-dependent surface markers that distinguish CSCs from their normal counterparts to enable selective targeting without impairing normal tissue regeneration.

## 5. Pathways and Their Roles in SSCs and CSCs

The role of signaling pathways in CSCs versus SSCs underscores how slight molecular shifts can have profound effects on cell behavior (Table 2). One such pathway, the Wnt/β-catenin signaling pathway, is crucial for regulating stem cell pluripotency and differentiation in SSCs, maintaining tissue homeostasis and regeneration by controlling the balance between self-renewal and differentiation [27]. However, in CSCs, Wnt pathway dysregulation fosters continuous stemness and unchecked proliferation, often inducing EMT, which escalates metastatic potential and chemotherapy resistance [61,62,63,64]. Targeting Wnt in CSCs could reduce their metastatic potential and augment the efficacy of standard therapeutics. However, this approach requires careful modulation to avoid adverse effects on SSCs, where Wnt signaling is essential for tissue repair and regeneration [63].

Similar to Wnt, the Notch signaling pathway plays a dual role in determining cell fate and maintaining SSC differentiation [27]. In SSCs, Notch activation is responsible for promoting differentiation processes that contribute to tissue integrity and repair. In CSCs, Notch signaling is often hyperactivated, promoting self-renewal and increasing tumorigenicity [63,65,66,67]. The overactivation of Notch signaling helps maintain CSC populations and supports tumor growth and resistance to therapies; consequently, therapeutic targeting of Notch signaling in CSCs may help reduce tumorigenicity and inhibit self-renewal. Modulating Notch activity could reduce tumorigenic capabilities, but once again, care must be taken to avoid impairing normal SSC function that relies on Notch signaling for differentiation and tissue repair [63].

Distinctively, Hedgehog signaling is pivotal in maintaining stem cell properties in SSCs through spatial and developmental patterning, regulating processes like proliferation and differentiation that are necessary for development and tissue regeneration [27]. The Hedgehog pathway is frequently dysregulated in CSCs, repurposing its organizational role to maintain malignant populations and promote resistance to chemotherapy [63,68]. In pancreatic cancer, inhibition of Hedgehog signaling has been shown to prevent EMT and tumor metastasis [69]. Targeting Hedgehog signaling in CSCs could help disrupt their maintenance and improve treatment response. The challenge lies in ensuring that normal stem cell functions, regulated by Hedgehog signaling, remain intact during treatment to avoid adverse effects on healthy tissue [63,68].

Complementing these pathways, TGF-β is involved in a large family of signaling processes that are essential for maintaining a balance between growth and differentiation in SSCs. TGF-β signaling allows SSCs to self-renew for tissue repair or differentiate into specialized cells as needed, playing a role in tissue homeostasis and wound healing [70,71]. In CSCs, TGF-β signaling is often dysregulated and hijacked to support tumor progression. Under hypoxic conditions in the tumor microenvironment, TGF-β signaling can induce EMT, enhance CSC plasticity, and promote invasion and metastasis [70,72]. It also enhances the expression of HIFs (HIF1α and HIF2α), contributing to aggressive tumor behavior and poor prognosis [73]. Thus, the TGF-β pathway represents a dual-edged sword in cancer therapy—while essential for tissue maintenance in SSCs, its dysregulation in CSCs makes it an attractive therapeutic target. Targeting specific components of the TGF-β/HIF axis could selectively inhibit CSC growth and prevent metastasis while preserving its beneficial roles in SSCs, providing a promising direction for novel cancer therapeutics [28].

EMT plays a central role in the emergence, maintenance, and functional properties of CSCs. EMT is a reversible process by which epithelial cells lose polarity and adhesion, acquiring mesenchymal traits such as motility, invasiveness, and resistance to apoptosis. This transition is primarily orchestrated by Wnt/β-catenin and TGF-β signaling, which converge on a set of EMT-associated transcription factors (EMT-TFs), including Snail, Twist, and ZEB1/2 [74]. In the canonical Wnt pathway, nuclear β-catenin activates EMT-TFs and promotes stem-like traits, while TGF-β signaling represses epithelial markers and induces mesenchymal gene expression [75,76]. These EMT-TFs not only drive cellular plasticity but also directly reprogram cancer cells into stem-like states, enhancing self-renewal and tumor-initiation potential. EMT and CSCs together promote immune evasion through altered antigen presentation and upregulation of immune checkpoint molecules, while also conferring resistance to chemotherapy and radiation via enhanced DNA repair, drug efflux, and anti-apoptotic signaling [76,77,78]. This EMT-CSC axis contributes to metastasis, therapeutic failure, and tumor recurrence, representing a critical therapeutic target in aggressive cancers [79].

## 6. Key Regulatory Transcription Factors

Beyond signaling pathways, the transcriptional landscape of CSCs and SSCs is further shaped by transcription factors like Oct4, Sox2, c-Myc, YAP/TAZ, and Nanog (Table 3). Each factor supports the natural stemness of SSCs, yet is co-opted in CSCs to drive malignancy, making them fundamental therapeutic targets [26].

### 6.1. Oct4

In SSCs, Oct4 (POU5F1) plays a crucial role in maintaining pluripotency and regulating the balance between self-renewal and differentiation. In iPSCs, Oct4 reprograms somatic cells back into a pluripotent state [80]. In CSCs, Oct4 is often overexpressed, which has been specifically linked to self-renewal in oral squamous cell carcinomas, pancreatic cancer, and gliomas, and further promotes tumor metastasis in lung cancer [81,110,111,112]. In cervical, hepatocellular, and lung cancer, high levels of Oct4 are associated with the activation of EMT signals, directly contributing to metastatic spread and therapeutic resistance [82,83,84,85]. Knockdown of Oct4 in pancreatic cancer has been shown to significantly reduce CSC malignancy [86]. Targeting Oct4 in CSCs could reduce their stemness and metastatic potential, providing a novel therapeutic approach to curtail tumor growth and improve treatment outcomes [86].

### 6.2. Sox2

Sox2 serves a comparable role, although it is specifically linked to ectodermal differentiation and CNS development in SSCs [87]. In SSCs, its expression helps balance stemness and differentiation, which are key to normal tissue maintenance. Elevated Sox2 expression is found in breast cancer cells, promoting sphere formation and proliferation, with additional evidence demonstrating its role in CSC resistance to tamoxifen [88,89]. Deletion of Sox2 has additionally been shown to prevent tumor initiation and impair CSC proliferation [90]. Further studies have demonstrated a role for Sox2 in maintaining the bidirectional transition of CSCs between stem-like and differentiated states [91,92,93]. Therefore, targeting Sox2 in CSCs could push cells towards terminal differentiation and diminish chemoresistance, making them more susceptible to standard cancer treatments [88,89].

### 6.3. C-Myc

In SSCs, c-Myc is a critical regulator of cell cycle progression, promoting the transition from the G1 to the S phase. It is essential for cellular proliferation and differentiation in various stem cell types [94]. In CSCs, c-Myc is an oncogene that drives the metabolic reprogramming necessary for survival in a nutrient-deprived tumor microenvironment. Overexpression of c-Myc leads to increased glucose and glutamine metabolism, supporting the rapid cell division and survival of CSCs [61]. This metabolic flexibility allows CSCs to adapt under conditions where normal cells would struggle to survive. Targeting c-Myc in CSCs has the potential to disrupt essential metabolic pathways, reducing their ability to proliferate and survive. One promising avenue involves targeting glutamine metabolism, as c-Myc-driven cancers frequently exhibit a strict dependency on glutamine [95]. Although this strategy could selectively weaken CSCs while minimizing adverse effects, it nevertheless requires careful targeting, as c-Myc also plays a vital role in normal stem cell proliferation and metabolism [96].

### 6.4. YAP/TAZ

Yes-associated protein (YAP) and transcriptional coactivator with PDZ-binding motif (TAZ) underscore the unique role of mechanical responsiveness, acting as transcriptional coactivators that support tissue regeneration by regulating stem cell proliferation and differentiation in response to mechanical cues in SSCs. YAP/TAZ activity is essential for the proliferation of MSCs, allowing them to differentiate into bone or cartilage in response to mechanical stress [97]. YAP and TAZ also help regulate epithelial stem cells in the skin and gut, maintaining a balance between self-renewal and differentiation for tissue integrity and repair [98]. In CSCs, YAP/TAZ signaling drives the expression of genes involved in cell proliferation, survival, and invasion. In the liver, YAP/TAZ signaling has been shown to induce the de-differentiation of adult hepatocytes, contributing to the generation and accumulation of CSCs [99]. YAP and TAZ also regulate genes involved in the extracellular matrix and cellular adhesion, creating a niche that sustains CSC survival and plasticity [31]. Inhibitors designed to prevent YAP/TAZ activation may hinder CSC survival by interfering with their ability to interact with the tumor microenvironment, thus enhancing the efficacy of existing therapies.

### 6.5. KLF4

KLF4 plays a dual role in SSCs, maintaining the balance between self-renewal and differentiation as well as helping stem cells maintain pluripotency. In breast and colorectal cancers, KLF4 has been shown to interact with other key transcription factors like Sox2 and Oct4 to regulate the balance between self-renewal and differentiation, thereby influencing tumor progression and response to therapy [28]. In osteosarcoma, glioma, and pancreatic cancer, however, KLF4 promotes self-renewal and sphere formation in CSCs via activation of the MAPK signaling pathway [100,101,102]. The ability of CSCs to prevent ubiquitination of KLF4, and thus enhance tumor metastasis, is a specific target for future therapeutic intervention [103,104].

### 6.6. Nanog

Nanog, a master regulator of pluripotency, maintains SSC self-renewal, yet is overexpressed in CSCs where it perpetuates self-renewal and aggressive tumor growth, especially in glioblastoma (GBM) and non-small cell lung cancer (NSCLC) [26,105]. In NSCLC, Nanog promotes CSC-associated tumor metastasis via downstream signaling pathways and protein expression that trigger the EMT process [30]. Inhibition of Nanog has been shown to reduce CSC self-renewal and impair their ability to drive tumor growth, positioning it as a promising target for cancer therapy [105].

### 6.7. SALL4

SALL4, a zinc finger transcription factor, is essential for maintaining pluripotency and self-renewal in SSCs such as hematopoietic stem cells but is typically silenced in differentiated cells. In CSCs, however, SALL4 is aberrantly re-expressed and activates oncogenic signaling pathways including Wnt/β-catenin, Notch, and PI3K/AKT, while promoting expression of key stemness-associated genes such as BMI1 and LIN28B. It also contributes to chemoresistance and immune evasion through epigenetic modulation of gene expression. Given its restricted expression in adult tissues, targeting SALL4 may selectively impair CSCs while sparing normal somatic stem cells [106,107].

### 6.8. FOXM1

FOXM1 is a cell cycle-associated transcription factor that regulates mitotic progression, DNA repair, and oxidative stress response. FOXM1 is active in proliferating SSCs and during tissue regeneration, but in CSCs, its sustained expression drives tumorigenicity, self-renewal, and resistance to therapy. FOXM1 also cooperates with other stemness-associated pathways to maintain the CSC phenotype. Pharmacologic or genetic inhibition of FOXM1 reduces CSC viability and sensitizes tumors to chemotherapy, with ongoing research into small-molecule inhibitors and RNA-based therapeutics [108].

### 6.9. EMT-Associated Transcription Factors

EMT-TFs, including Snail, Slug, Twist, ZEB1, and ZEB2, play central roles in plasticity and reprogramming. In SSCs, these factors are transiently activated during development or repair processes. In CSCs, however, persistent EMT-TF expression promotes repression of epithelial markers such as E-cadherin, activation of mesenchymal traits, and induction of stem-like programs, enabling metastasis, immune evasion, and therapeutic resistance. These EMT-TFs are tightly regulated by post-translational modifications that fine-tune their stability and activity, reinforcing the invasive phenotype of CSCs. While direct pharmacological targeting of these factors remains challenging, modulating their upstream regulators (e.g., E3 ubiquitin ligases, signaling pathways) or interfering with their post-translational modifications is a promising strategy under investigation [79,109]. These transcriptional regulators, collectively, offer a suite of promising targets within a precision oncology framework.

## 7. Differences in Transcription Factor Regulation

**Figure 1:**
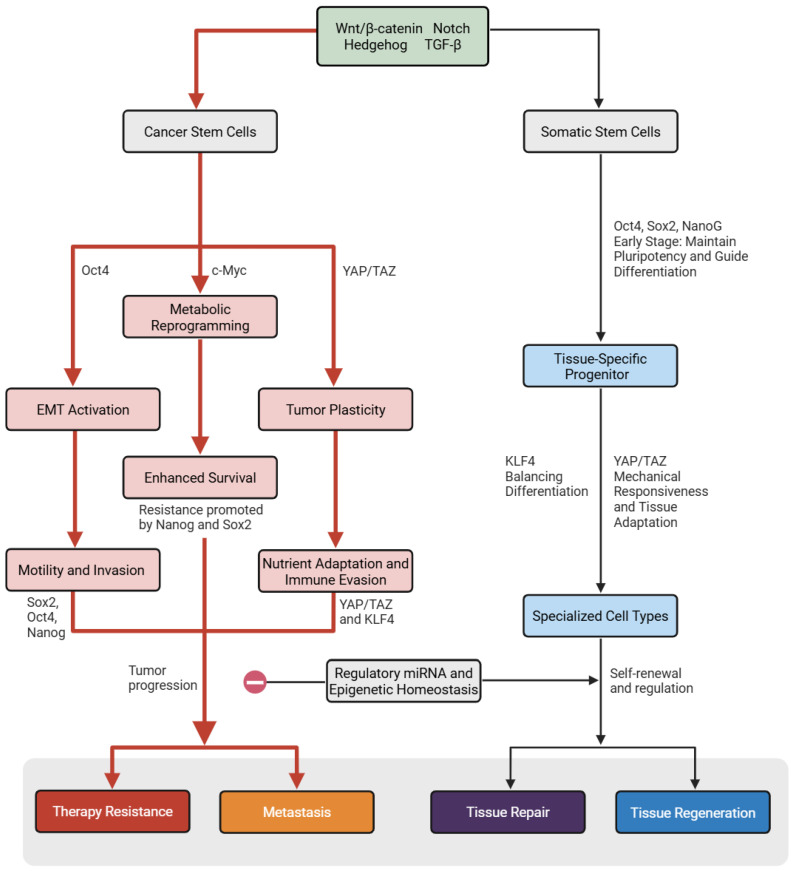
Divergent regulation of signaling pathways in SSCs versus CSCs. This schematic compares the signaling environments and regulatory states of SSCs (left) and CSCs (right). In SSCs, pathways such as Wnt/β-catenin, Notch, Hedgehog, and TGF-β are tightly controlled to maintain homeostasis, with balanced expression of stemness transcription factors (Oct4, Sox2, Nanog, c-Myc, YAP/TAZ), restricted chromatin accessibility at pluripotency loci, active tumor-suppressive microRNAs (e.g., let-7, miR-34a), and negative feedback from differentiated progeny. In contrast, CSCs exhibit pathway hyperactivation, sustained expression of pluripotency factors, open and bivalent chromatin states, and loss of regulatory microRNAs, enabling epithelial-mesenchymal transition (EMT), immune evasion, therapy resistance, and unchecked self-renewal. These distinctions illustrate how CSCs hijack normal stem cell signaling to support tumor initiation, progression, and relapse. Red arrows indicate aberrant, hyperactivated processes and downstream oncogenic consequences, black arrows indicate controlled physiologic differentiation and self-renewal pathways, and the prohibition sign denotes the absence of regulation by microRNAs and epigenetic homeostasis in CSCs.

Despite sharing core transcription factors such as Oct4 and TGF-β, SSCs and CSCs diverge markedly in function due to context-specific regulation (Figure 1). In SSCs, factors like Oct4 and TGF-β (including Nodal) signaling maintain self-renewal in a tightly controlled manner, with chromatin at pluripotency loci (e.g., Oct4, Nanog) epigenetically repressed and selectively accessible to SSC-specific transcription factors [113,114]. In CSCs, these same pathways are aberrantly activated: Oct4 is overexpressed, driving dedifferentiation, chemoresistance, and immune evasion, while TGF-β signaling induces EMT and enhances stemness through both Smad-dependent and independent mechanisms [115,116].

These differences are reinforced by epigenomic and post-transcriptional factors. CSCs exhibit globally increased chromatin accessibility and bivalent histone marks that enable transcriptional plasticity [117]. Tumor-suppressive microRNAs (e.g., let-7, miR-34a) are often downregulated, while oncogenic microRNAs (miRNAs) promote pluripotency gene expression [118]. Finally, CSCs escape negative feedback loops that normally constrain SSC proliferation, allowing for unchecked self-renewal and tumor propagation [119,120]. These dysregulated layers of control, rather than the presence of stemness genes themselves, distinguish CSC behavior from normal stem cell function.

## 8. Epigenetic Modifications

Epigenetic modifications are another layer of regulation that distinguishes CSCs from SSCs (Table 4). In SSCs, DNA methylation and histone modifications play key roles in regulating normal tissue homeostasis and differentiation by switching genes on or off in response to environmental signals. This ensures proper cell fate determination and prevents aberrant growth. However, in CSCs, these epigenetic modifications become dysregulated, often leading to the silencing of tumor suppressor genes and activation of oncogenes that support malignant behavior [26,121]. For example, DNA methylation-induced silencing of p16 and Apc genes, crucial for stem cell self-renewal, has been observed in several cancers [122]. Recent research further indicates that DNA demethylation can contribute to CSC resistance to specific cancer therapies, including sorafenib [123]. Specific histone modifications, such as H3K4me-dependent methylation, have also been linked to therapy resistance, notably in ovarian cancer where it promotes platinum resistance through elevated GALNT10 gene expression [124,125].

Beyond these modifications, large-scale regulation of chromatin architecture plays a pivotal role in stem cell biology. Chromatin remodeling complexes orchestrate the dynamic balance between open and closed chromatin states, governing access to lineage-specific transcriptional programs. In SSCs, the SWI/SNF family of ATP-dependent chromatin remodelers facilitates nucleosome repositioning to permit gene activation, while Polycomb group (PcG) complexes such as PRC2 enforce transcriptional silencing through H3K27 trimethylation. This interplay establishes a poised, bivalent chromatin configuration that allows SSCs to respond rapidly to differentiation signals [126,127]. In CSCs, this regulatory balance is frequently disrupted. Loss or mutation of SWI/SNF subunits, which has been detected in approximately 25% of human cancers, can impair lineage commitment, enable cellular plasticity, and reinforce malignant transcriptional programs. Conversely, unopposed Polycomb activity may silence differentiation or tumor suppressor genes, supporting CSC self-renewal and therapeutic resistance [126,127,128,129].

Other epigenetic regulators further reinforce CSC identity. SETD1A, a histone methyltransferase of the COMPASS complex, catalyzes H3K4 methylation and activates stemness-associated enhancers in hepatocellular carcinoma stem cells [136]. BRD4, a bromodomain-containing epigenetic reader, binds acetylated histones and promotes transcriptional elongation at oncogenic loci and super-enhancers in squamous cell carcinomas [137]. In SSCs, SETD1A and BRD4 support normal stem cell proliferation and cell cycle progression; in CSCs, their aberrant activity drives persistent expression of pluripotency, survival, and chemoresistance genes [136,137]. Collectively, the disruption of epigenetic homeostasis in CSCs fosters plasticity, immune evasion, and treatment failure, altogether positioning epigenetic modification as a key target area for restoring transcriptional control and therapeutic vulnerability [130].

## 9. Non-Coding RNA

In addition to epigenetic markers, non-coding RNAs, such as miRNAs and exosomes, play pivotal roles in CSC function and therapy resistance (Table 4). miRNAs are frequently dysregulated in CSCs, contributing to stemness, therapy resistance, and metastatic potential. For instance, miR21, an onco-miRNA, is upregulated in several cancers, leading to the inhibition of tumor suppressor genes like PTEN, thereby enhancing CSC proliferation and survival [26,131,132]. Similarly, exosomes play a crucial role in cell–cell communication and can transfer oncogenic signals between CSCs and their microenvironment. The distinct exosomal miRNA profiles of CSCs can influence the behavior of surrounding cells, promoting an oncogenic environment that supports cancer progression and both local and distant metastasis [85,133,134,135]. By focusing on the unique miRNA and exosome profiles of CSCs, researchers can develop targeted therapies that selectively inhibit these cancer-promoting signals [138]. For example, a preclinical study demonstrated that targeting exosomal miR-183-5p in colorectal cancer suppressed angiogenesis and metastatic progression by inhibiting FOXO1 expression [134]. Such approaches hold promise for improving the specificity and efficacy of cancer treatments while simultaneously minimizing damage to normal stem cells, as well as for predicting metastatic propensity and preventing organ-specific metastasis [135]. 

## 10. Implications for Cancer Therapy

In exploring the transcriptomic similarities and differences between SSCs and CSCs, we provided a pathway-centered analysis that could inform therapeutic strategies. Both CSCs and SSCs share hallmark features of stemness, including self-renewal and differentiation capabilities. However, CSCs exhibit distinct transcriptomic profiles that confer aggressive behaviors such as uncontrolled proliferation, metastasis, and resistance to traditional chemotherapy [139,140]. Pathways like Wnt, Notch, Hedgehog, TGF-β, and transcription factors like Oct4, Sox2, c-Myc, YAP/TAZ, Nanog, and KLF4, although vital for stem cell function, become dysregulated in CSCs, leading to uncontrolled growth, metastasis, and resistance to therapies [139,140,141,142]. Nevertheless, the intricate relationship between stem cells and cancer cells provides a unique opportunity for therapeutic intervention in oncology despite the cellular, genetic, and epigenetic similarities between SSCs and CSCs [141,142]. Targeting CSC cell surface markers, transcription factors, signaling pathways, metabolic processes, and epigenetic modifications, as well as overlying regulatory networks, has the potential to revolutionize cancer therapy [26,27,142]. Still, effective treatments remain elusive due to the inherent adaptive nature of CSCs and their ability to differentiate, de-differentiate, and enter a quiescent state, all in the context of the inherently supportive CSC niche [139,143,144,145].

The translation of stem cell knowledge into therapeutic strategies has to date primarily focused on targeting specific cell surface markers, but there is significant potential to expand therapies by addressing transcription factors, signaling pathways, metabolic processes, and epigenetic modifications [145,146]. So far, therapies targeting cell surface markers such as CD123, CD44v6, and EpCAM have shown promise in eliminating CSCs and reducing the potential for tumor relapse [52]. Pivekimab sunirine (IMGN632), for instance, has shown promise in targeting CD123 to treat conditions like refractory acute myeloid leukemia and plasmacytoid dendritic cell tumors, with initial data supporting a breakthrough therapy designation from the FDA [147,148].

Despite these advances, translation to clinical efficacy remains challenging due to the shared expression of many target pathways between CSCs and SSCs. Clinical trials have further underscored the delicate balance required to suppress CSCs without compromising SSC-mediated tissue repair and regeneration. Pathway-targeted agents—such as Wnt inhibitors (e.g., vantictumab), Notch pathway antagonists (e.g., demcizumab, gamma-secretase inhibitors), and Hedgehog inhibitors—have been limited by on-target/off-tumor toxicities affecting normal stem cell compartments like the intestinal crypt or bone marrow [149]. However, exceptions have been noted with the approval of the first Hedgehog pathway inhibitors, vismodegib and sonidegib, to treat basal cell carcinoma [150]. Still, the intratumoral heterogeneity and plasticity of CSC populations enable dynamic resistance to monotherapies, further emphasizing the need for combination regimens and precision-guided interventions. Concurrently, several therapies targeting transcription factors like Oct4, Sox2, and Nanog, as well as treatments targeting epigenetic modifications, are in early-phase clinical trials aiming to disrupt core CSC regulatory networks while minimizing collateral damage to SSCs [26,52].

Pancreatic ductal adenocarcinoma (PDAC) serves as a well-studied model for investigating CSC biology, offering extensive in vivo and translational evidence that links CSC programs to tumor initiation, progression, and therapeutic resistance. Foundational xenograft work identified a CD44+/CD24+/ESA+ CSC subpopulation with robust tumor-initiating capacity and self-renewal; as few as ~100 cells formed tumors and showed elevated Sonic Hedgehog expression, establishing CSC linkage to developmental signaling and tumor initiation in vivo [151,152]. Subsequent patient-derived xenograft (PDX) and genetically engineered mouse models (GEMMs) connected CSC programs to EMT, metastatic dissemination, and chemoresistance and also situated CSCs within the desmoplastic, immunosuppressive stroma that impedes drug delivery and shapes survival [153,154]. These CSC-enriched populations exhibit elevated Hedgehog activity, and inhibition of this pathway in PDX and GEMMs transiently reduces CSC frequency, decreases stromal density, enhances intratumoral gemcitabine delivery, and suppresses metastasis, particularly when combined with agents targeting CXCR4-mediated stromal signaling [155,156]. Clinical trials targeting this axis, however, underscore translational challenges. In patients with metastatic PDAC, vismodegib suppressed GLI1/PTCH1 but did not reduce CSCs, improve progression-free or overall survival, or outperform gemcitabine, illustrating pathway modulation without clinical benefit and potential compensation via noncanonical GLI activation and stromal reprogramming [157,158]. TGF-β inhibitors similarly remain investigational. Preclinical rationale supports combination with cytotoxic medications or immunotherapy to target CSC-stroma circuits, but the definitive survival benefit of TGF-β inhibition in PDAC has not yet been firmly established [159,160].

Glioblastoma represents another malignancy in which CSC biology has been extensively characterized, with in vivo and clinical studies demonstrating the role of CSCs in tumor maintenance, therapeutic resistance, and recurrence. The CSC paradigm in GBM was established when CD133+ fractions of patient-derived tumor cells reproducibly initiated tumors after orthotopic implantation into mouse brains, faithfully recapitulating parental tumor histology [161]. Subsequent PDX research linked glioma stem cells (GSCs) to pathway activation (e.g., Notch, Wnt/β-catenin), enhanced DNA repair, and therapy resistance, which enables tumor recurrence [162]. In GBM neurospheres and xenografts, Notch blockade via gamma-secretase inhibitors (GSIs) depleted CD133+ cells, reduced clonogenicity, suppressed xenograft growth, and prolonged survival, with decreased AKT/STAT3 signaling. Genetic Notch activation demonstrated opposite effects [163]. Early clinical experiences with GSIs such as MRK003 and RO4929097 in GBM have shown pathway modulation and acceptable safety but no consistent, durable efficacy, reflecting pathway redundancy and blood-brain barrier constraints [164,165,166,167,168]. Wnt/β-catenin is similarly implicated in GSC maintenance and CD133-driven programs, but clinical translation of Wnt inhibitors in GBM remains preliminary [169,170]. Parallel differentiation therapy efforts have leveraged bone morphogenic protein 4 (BMP4) to drive GSCs toward less tumorigenic fates. BMP4 promotes GSC differentiation and depletes stem-like states preclinically, reducing tumorigenicity, although clinical validation is nascent [171,172]. These findings have also established a foundation for combination strategies, such as BMP4 with temozolomide, informed by patient-derived models [173].

The insights gained from SSC biology inform these strategies, as many mechanisms that govern SSC self-renewal, differentiation, and plasticity are mirrored or co-opted in CSCs. For example, understanding the regulatory roles of transcription factors like Oct4 and Sox2 in maintaining SSC pluripotency has directly shaped approaches to disrupting their analogous functions in CSCs [86,88]. Similarly, insights into the metabolic and epigenetic regulation of SSCs are providing a foundation for developing CSC-targeted therapies that minimize off-target effects on normal stem cells [26,146]. By leveraging the parallels between SSCs and CSCs, therapeutic strategies can be designed not only to eradicate CSCs but also to preserve normal tissue homeostasis. Difficulties remain, however, in isolating the activity of these novel cancer therapeutics from the tumor microenvironment and regulatory networks that constantly modify and protect CSCs from existing cancer drugs [27,52].

Given this multifactorial pathogenesis, precision medicine will become increasingly important for cancer patients in the coming decades. Leveraging transcriptomic data can pinpoint specific mechanisms underlying CSC behavior; by targeting the unique signaling pathways and transcriptional networks that sustain CSCs, researchers can design more effective, personalized therapies. Bulk RNA sequencing further masks cell heterogeneity; the expanding use of scRNA-seq and spatial transcriptomics is revolutionizing current understanding of stem cell plasticity, lineage trajectories, and tumor microenvironments [174]. The computational tools of precision medicine include Bayesian inference, big-data simulations of microenvironment interactions, N-of-1 clinical trials, and predictive epigenetic modeling. Bayesian inference integrates genomic and transcriptomic data to predict personalized treatment responses, while big-data simulations model how CSCs interact with their microenvironment, helping to identify resistance mechanisms. N-of-1 clinical trials allow for personalized therapies tailored to the heterogeneity of CSC-driven cancers, and predictive epigenetic modeling offers insights into disrupting CSC-specific gene regulation [7,175]. Together, these techniques provide a framework for designing highly targeted and effective therapies.

Despite advances in transcriptomic profiling and targeted interventions, it is essential to recognize that the defining features of both SSCs and CSCs, including self-renewal, differentiation, and niche interactions, are executed within a complex in vivo environment. As such, tools like lineage tracing, spatial transcriptomics, PDX models, and organoid models are increasingly important for validating stem cell behaviors and therapeutic responses in a physiologically relevant context. These models bridge the gap between molecular insights and clinical application, helping to refine CSC-targeted therapies while preserving SSC function. Incorporating such in vivo strategies into future studies will be crucial for translating transcriptomic discoveries into durable and effective cancer treatments.

Ultimately, the integration of transcriptomic analyses into cancer research holds promise for revolutionizing how we approach cancer treatment. Continued integration of transcriptomics in cancer research could unravel the complex molecular landscapes of CSCs and SSCs, leading to innovative therapies that improve patient outcomes and provide durable remission in oncology.

## 11. Conclusions

Somatic stem cells (SSCs) and cancer stem cells (CSCs) share core regulatory pathways and transcription factors that govern stemness, self-renewal, and plasticity. However, CSCs hijack these mechanisms through aberrant signaling, epigenetic reprogramming, and microenvironmental adaptation in order to drive malignancy, metastasis, and resistance to therapy. This review integrates current transcriptomic and epigenetic evidence to delineate the divergent regulatory landscapes of SSCs and CSCs, with a focus on chromatin architecture, transcription factor networks, and cell surface signaling regulators. We also present a comparative framework to highlight how CSC-specific dysregulation enables immune evasion and therapeutic resistance.

By consolidating findings across developmental biology, cancer genomics, and stem cell epigenetics, this synthesis informs precision oncology efforts to selectively target CSC vulnerabilities while preserving normal stem cell function. Emerging insights into chromatin modulators, microRNAs, and surface markers underscore the potential for next-generation therapies aimed at CSC eradication. Continued integration of single-cell transcriptomics, epigenomic profiling, and functional assays will be essential to resolve intratumoral heterogeneity and translate molecular understanding into effective, durable cancer treatments. As such, this review provides a cross-disciplinary resource for both regenerative medicine and cancer biology, bridging fundamental stem cell science with translational oncology.

## Figures and Tables

**Table 1:** Cell surface regulators in SSCs and CSCs.

Cell Surface Regulator	Type	Role in SSCs	Role in CSCs
CD133	Pentaspan membrane glycoprotein	- Marker of neural and hematopoietic stem cells; involved in membrane organization [48,49].	- CSC marker in glioblastoma, liver, and prostate cancers; enhances tumor-initiating capacity and survival [48,49].
CD44	Adhesion receptor	- Mediates stem cell homing and adhesion to the niche; interacts with ECM components [50,51].	- Marks CSCs in breast, colon, and head/neck cancers; promotes EMT, metastasis, and chemoresistance [50,51].
EpCAM	Cell adhesion molecule	- Supports epithelial stem cell proliferation and cell–cell adhesion [52,53].	- Overexpressed in CSCs from colorectal and pancreatic tumors; regulates proliferation and immune evasion [52,53].
CD25	Cytokine receptor subunit	- Expressed on activated immune and progenitor cells; regulates immune and stem signaling [54].	- Aberrantly expressed in some CSC populations; implicated in leukemogenesis and immune modulation [54].
LGR5	G-protein coupled receptor	- Wnt target gene; maintains intestinal and hair follicle stem cells [55].	- CSC marker in colon and liver cancer; supports Wnt-driven tumorigenesis and resistance [55].
ICAM1 (CD54)	Immunoglobulin superfamily	- Facilitates stem cell adhesion and immune interaction; expressed in endothelial SSCs [56].	- Enhances CSC immune evasion, extravasation, and metastasis; upregulated in aggressive cancers [56].
ALDH1	Detoxifying enzyme	- Marks quiescent SSCs with high self-renewal; detoxifies aldehydes to prevent damage [57].	- High ALDH activity defines CSCs across tumors; mediates resistance to chemotherapy and oxidative stress [57].
CD34	Cell surface glycoprotein	- Classical marker for hematopoietic and endothelial progenitor cells [58].	- Marks CSC subsets in leukemia and solid tumors; role in adhesion and microenvironmental signaling [58].
Integrin α6/β1	ECM receptor (Integrin family)	- Anchors SSCs to niche matrix proteins like laminin; regulates quiescence [59].	- Supports CSC invasion and interaction with tumor stroma; linked to radiation resistance in gliomas [59].
Notch Receptors	Transmembrane signaling receptor	- Mediates cell fate decisions and lateral inhibition in developing tissues [60].	- Aberrant activation sustains CSC self-renewal, especially in breast and brain tumors [60].

**Table 2:** Key signaling pathways in SSCs and CSCs.

Signaling Pathway	Role in SSCs	Role in CSCs	Therapeutic Potential
Wnt/β-catenin	- Regulates stem cell pluripotency and differentiation to maintain tissue homeostasis [27].	- Hyperactivation fosters continuous stemness, induces EMT, increases metastatic potential, and drives chemotherapy resistance [61,62,63,64].	- Targeting Wnt signaling in CSCs could reduce their metastatic potential and improve therapeutic response [63].
Notch	- Promotes differentiation and tissue integrity in SSCs [27].	- Hyperactivated Notch signaling in CSCs supports self-renewal, tumor growth, and resistance to therapies [65,66,67].	- Notch inhibitors could reduce tumorigenicity while preserving SSC function [66,67].
Hedgehog	- Regulates proliferation and differentiation, critical for development and tissue regeneration [27].	- Frequently dysregulated in CSCs, sustaining malignant populations and promoting resistance to chemotherapy [68,69].	- Hedgehog inhibition has been shown to prevent EMT and metastasis in pancreatic cancer [69].
TGF-β	- Supports self-renewal or differentiation depending on tissue needs [70,71].	- Hijacked by CSCs under hypoxic conditions to drive EMT, enhance CSC plasticity, increase the expression of HIFs, and promote metastasis [70,72,73].	- Targeting specific components of the TGF-β/HIF axis may inhibit CSCs and reduce metastatic potential while preserving SSC function [73].

**Table 3:** Key transcriptomic factors in SSCs and CSCs.

Transcription Factor	Role in SSCs	Role in CSCs	Therapeutic Potential
Oct4 (POU5F1)	- Maintains pluripotency and regulates the balance between self-renewal and differentiation [80].	- Overexpressed in oral squamous cell carcinomas, pancreatic cancer, gliomas, and lung cancer, promoting self-renewal, EMT, metastasis, and therapeutic resistance [81,82,83,84,85].	- Knockdown studies in pancreatic cancer reduce CSC malignancy [86].
Sox2	- Balances stemness and differentiation, crucial for ectodermal differentiation and CNS development [87].	- Elevated expression in breast cancer enhances sphere formation, proliferation, and tamoxifen resistance [88,89]. - Maintains CSC plasticity between stem-like and differentiated states [90,91,92,93].	- Deletion prevents tumor initiation and CSC proliferation; targeting may induce differentiation and reduce chemoresistance [88,89].
c-Myc	- Regulates cell cycle progression, essential for proliferation and differentiation [94].	- Drives metabolic reprogramming to survive nutrient-deprived environments; overexpression increases glucose and glutamine metabolism [95].	- Targeting c-Myc disrupts metabolic pathways; glutamine metabolism is a specific vulnerability [95,96].
YAP/TAZ	- Responds to mechanical cues, regulating proliferation and differentiation in tissue regeneration [97,98].	- Activates genes for proliferation, survival, invasion, and niche maintenance; promotes CSC de-differentiation in the liver [31,99].	- Inhibitors of YAP/TAZ activation disrupt CSC survival and tumor microenvironment interactions [38,99].
KLF4	- Maintains balance between self-renewal and differentiation; helps sustain pluripotency [100,101].	- Promotes CSC sphere formation in osteosarcoma, glioma, and pancreatic cancer; prevents ubiquitination to enhance metastasis [100,101,102,103,104].	- Targeting KLF4’s role in CSCs reduces metastasis; strategies include interfering with MAPK signaling and preventing ubiquitination [100,101,102,103,104].
Nanog	- Master regulator of pluripotency, maintaining self-renewal [26,105].	- Overexpressed in glioblastoma and non-small cell lung cancer; promotes EMT, self-renewal, and aggressive tumor growth [26,30,105].	- Inhibition reduces CSC self-renewal and tumor growth; a promising target for therapy [30,105].
SALL4	- Critical for early hematopoietic and pluripotent SSC maintenance; silenced upon differentiation [106,107].	- Activates Wnt, Notch, and PI3K/AKT signaling; promotes stemness gene expression (BMI1, LIN28B), chemoresistance, and immune modulation [106,107].	- Inhibition impairs CSC growth while sparing normal SSCs; SALL4 is a selective target due to minimal adult tissue expression [106,107]
FOXM1	- Regulates cell cycle progression and regeneration in SSCs [108].	- Maintains CSC proliferation, self-renewal, DNA repair, and therapy resistance [108].	- Pharmacologic and genetic inhibition reduces CSC viability and sensitizes tumors to therapy [108].
EMT-TFs (Snail, Slug, Twist, ZEB1/2)	- Involved in developmental transitions and tissue repair; transient expression in SSCs [79,109].	- Induces EMT, plasticity, and stem-like reprogramming in CSCs; promotes metastasis, immune evasion, and resistance to therapy [79,109].	- Therapeutic approaches target upstream regulators and post-translational modifiers to disrupt EMT-TF-driven CSC programs [79,109].

**Table 4:** Epigenetic and non-coding RNA modifications in SSCs and CSCs.

Mechanism	Role in SSCs	Role in CSCs	Therapeutic Potential
DNA Methylation	- Regulates differentiation and normal tissue homeostasis [26,121].	- Hypermethylation in CSCs silences tumor suppressors (e.g., p16, Apc), enhancing malignancy [122,123].	- Reversing DNA methylation may restore tumor suppressor function, reducing CSC-driven progression [123].
Histone Modifications	- Controls gene expression via chromatin accessibility, ensuring proper cell differentiation [121].	- Aberrant histone modifications in CSCs support plasticity and therapy resistance [124,125].	- Histone deacetylase inhibition may reduce CSC survival and promote differentiation [124,125].
Chromatin Modifiers	- SWI/SNF, Polycomb, SETD1A, and BRD4 coordinate gene activation and repression for lineage control [126,127].	- Frequently dysregulated in CSCs; promote stemness, immune evasion, and resistance through chromatin remodeling and transcriptional rewiring [126,127,128,129].	- Targeting BRD4, SETD1A, or restoring SWI/SNF function may suppress CSC viability and restore differentiation [130].
MicroRNAs	- Fine-tune gene expression to regulate SSC differentiation and function [26,131].	- CSC-associated miRNAs (e.g., miR-21) suppress tumor suppressor genes, enhancing CSC proliferation and survival [131,132].	- Blocking oncogenic miRNAs (e.g., miR-21) could reduce CSC viability [132]
Exosomal Signaling	- Facilitates cell–cell communication for tissue repair [133].	- CSC-derived exosomes transfer oncogenic signals, promoting cancer progression and metastasis [133,134,135].	- Disrupting exosomal signaling in CSCs may suppress tumor growth and prevent both local and distant metastasis [134,135].

## Data Availability

Not applicable.

## References

[1] Loh J.J., Ma S. (2024). Hallmarks of cancer stemness. Cell Stem Cell.

[2] Eid R.A., Alaa Edeen M., Shedid E.M., Kamal A.S.S., Warda M.M., Mamdouh F., Khedr S.A., Soltan M.A., Jeon H.W., Zaki M.S.A. (2023). Targeting Cancer Stem Cells as the Key Driver of Carcinogenesis and Therapeutic Resistance. Int. J. Mol. Sci..

[3] Aponte P.M., Caicedo A. (2017). Stemness in Cancer: Stem Cells, Cancer Stem Cells, and Their Microenvironment. Stem Cells Int..

[4] Takahashi K., Yamanaka S. (2006). Induction of pluripotent stem cells from mouse embryonic and adult fibroblast cultures by defined factors. Cell.

[5] Gao F., Chiu S.M., Motan D.A., Zhang Z., Chen L., Ji H.L., Tse H.F., Fu Q.L., Lian Q. (2016). Mesenchymal stem cells and immunomodulation: Current status and future prospects. Cell Death Dis..

[6] Stavely R., Robinson A.M., Fraser S., Filippone R.T., Stojanovska V., Eri R., Apostolopoulos V., Sakkal S., Nurgali K. (2024). Bone marrow-derived mesenchymal stem cells mitigate chronic colitis and enteric neuropathy via anti-inflammatory and anti-oxidative mechanisms. Sci. Rep..

[7] Wang R., Yao Q., Chen W., Gao F., Li P., Wu J., Yu J., Cao H. (2021). Stem cell therapy for Crohn’s disease: Systematic review and meta-analysis of preclinical and clinical studies. Stem Cell Res. Ther..

[8] Zeng L., Yang K., Yu G., Chen J., Long Z., Xiang W., Liu S., Zheng Y., Yan Y., Hao M. (2024). Efficacy and safety of culture-expanded mesenchymal stromal cell therapy in the treatment of 4 types of inflammatory arthritis: A systematic review and meta-analysis of 36 randomized controlled trials. Semin. Arthritis Rheum..

[9] Zhidu S., Ying T., Rui J., Chao Z. (2024). Translational potential of mesenchymal stem cells in regenerative therapies for human diseases: Challenges and opportunities. Stem Cell Res. Ther..

[10] Menendez P., Wang L., Bhatia M. (2005). Genetic manipulation of human embryonic stem cells: A system to study early human development and potential therapeutic applications. Curr. Gene Ther..

[11] Schweitzer J.S., Song B., Herrington T.M., Park T.Y., Lee N., Ko S., Jeon J., Cha Y., Kim K., Li Q. (2020). Personalized iPSC-Derived Dopamine Progenitor Cells for Parkinson’s Disease. N. Engl. J. Med..

[12] (2021). First Parkinson’s patients dosed with dopaminergic neurons. Nat. Biotechnol..

[13] Piao J., Zabierowski S., Dubose B.N., Hill E.J., Navare M., Claros N., Rosen S., Ramnarine K., Horn C., Fredrickson C. (2021). Preclinical Efficacy and Safety of a Human Embryonic Stem Cell-Derived Midbrain Dopamine Progenitor Product, MSK-DA01. Cell Stem Cell.

[14] Loring J.F. (2018). Autologous Induced Pluripotent Stem Cell-Derived Neurons to Treat Parkinson’s Disease. Stem Cells Dev..

[15] (2023). Parkinson’s iPSC trial. Nat. Biotechnol..

[16] Vo Q.D., Saito Y., Nakamura K., Iida T., Yuasa S. (2024). Induced Pluripotent Stem Cell-Derived Cardiomyocytes Therapy for Ischemic Heart Disease in Animal Model: A Meta-Analysis. Int. J. Mol. Sci..

[17] Farboud S.P., Fathi E., Valipour B., Farahzadi R. (2024). Toward the latest advancements in cardiac regeneration using induced pluripotent stem cells (iPSCs) technology: Approaches and challenges. J. Transl. Med..

[18] Sugiura T., Shahannaz D.C., Ferrell B.E. (2024). Current Status of Cardiac Regenerative Therapy Using Induced Pluripotent Stem Cells. Int. J. Mol. Sci..

[19] Liu H., Huang S.S., Lingam G., Kai D., Su X., Liu Z. (2024). Advances in retinal pigment epithelial cell transplantation for retinal degenerative diseases. Stem Cell Res. Ther..

[20] Mandai M., Watanabe A., Kurimoto Y., Hirami Y., Morinaga C., Daimon T., Fujihara M., Akimaru H., Sakai N., Shibata Y. (2017). Autologous Induced Stem-Cell-Derived Retinal Cells for Macular Degeneration. N. Engl. J. Med..

[21] Ogi D.A., Jin S. (2023). Transcriptome-Powered Pluripotent Stem Cell Differentiation for Regenerative Medicine. Cells.

[22] Wu C.L., Dicks A., Steward N., Tang R., Katz D.B., Choi Y.R., Guilak F. (2021). Single cell transcriptomic analysis of human pluripotent stem cell chondrogenesis. Nat. Commun..

[23] Ciminieri C., Woest M.E., Reynaert N.L., Heijink I.H., Wardenaar R., Spierings D.C.J., Brandsma C.A., Königshoff M., Gosens R. (2023). IL-1β Induces a Proinflammatory Fibroblast Microenvironment that Impairs Lung Progenitors’ Function. Am. J. Respir. Cell Mol. Biol..

[24] Ramabadran R., Wang J.H., Reyes J.M., Guzman A.G., Gupta S., Rosas C., Brunetti L., Gundry M.C., Tovy A., Long H. (2023). DNMT3A-coordinated splicing governs the stem state switch towards differentiation in embryonic and haematopoietic stem cells. Nat. Cell Biol..

[25] Testa C., Oliveto S., Jacchetti E., Donnaloja F., Martinelli C., Pinoli P., Osellame R., Cerullo G., Ceri S., Biffo S. (2022). Whole transcriptomic analysis of mesenchymal stem cells cultured in Nichoid micro-scaffolds. Front. Bioeng. Biotechnol..

[26] Zeng Z., Fu M., Hu Y., Wei Y., Wei X., Luo M. (2023). Regulation and signaling pathways in cancer stem cells: Implications for targeted therapy for cancer. Mol. Cancer.

[27] Rossi F., Noren H., Jove R., Beljanski V., Grinnemo K.H. (2020). Differences and similarities between cancer and somatic stem cells: Therapeutic implications. Stem Cell Res. Ther..

[28] Ayob A.Z., Ramasamy T.S. (2018). Cancer stem cells as key drivers of tumour progression. J. Biomed. Sci..

[29] Beck B., Blanpain C. (2013). Unravelling cancer stem cell potential. Nat. Rev. Cancer.

[30] Liu Q., Guo Z., Li G., Zhang Y., Liu X., Li B., Wang J., Li X. (2023). Cancer stem cells and their niche in cancer progression and therapy. Cancer Cell Int..

[31] Jokela T.A., LaBarge M.A. (2021). Integration of mechanical and ECM microenvironment signals in the determination of cancer stem cell states. Curr. Stem Cell Rep..

[32] Clevers H. (2011). The cancer stem cell: Premises, promises and challenges. Nat. Med..

[33] Mannino G., Russo C., Maugeri G., Musumeci G., Vicario N., Tibullo D., Giuffrida R., Parenti R., Lo Furno D. (2022). Adult stem cell niches for tissue homeostasis. J. Cell Physiol..

[34] Goodell M.A., Nguyen H., Shroyer N. (2015). Somatic stem cell heterogeneity: Diversity in the blood, skin and intestinal stem cell compartments. Nat. Rev. Mol. Cell Biol..

[35] Sneddon J.B., Werb Z. (2007). Location, location, location: The cancer stem cell niche. Cell Stem Cell.

[36] Kraiczy J., McCarthy N., Malagola E., Tie G., Madha S., Boffelli D., Wagner D.E., Wang T.C., Shivdasani R.A. (2023). Graded BMP signaling within intestinal crypt architecture directs self-organization of the Wnt-secreting stem cell niche. Cell Stem Cell.

[37] Maimets M., Pedersen M.T., Guiu J., Dreier J., Thodberg M., Antoku Y., Schweiger P.J., Rib L., Bressan R.B., Miao Y. (2022). Mesenchymal-epithelial crosstalk shapes intestinal regionalisation via Wnt and Shh signalling. Nat. Commun..

[38] Ormsbee Golden B.D., Gonzalez D.V., Yochum G.S., Coulter D.W., Rizzino A. (2024). SOX2 represses c-MYC transcription by altering the co-activator landscape of the c-MYC super-enhancer and promoter regions. J. Biol. Chem..

[39] Fu R.Z., Cottrell O., Cutillo L., Rowntree A., Zador Z., Wurdak H., Papalopulu N., Marinopoulou E. (2024). Identification of genes with oscillatory expression in glioblastoma: The paradigm of SOX2. Sci. Rep..

[40] Habib S.J., Chen B.C., Tsai F.C., Anastassiadis K., Meyer T., Betzig E., Nusse R. (2013). A localized Wnt signal orients asymmetric stem cell division in vitro. Science.

[41] Goldstein B., Takeshita H., Mizumoto K., Sawa H. (2006). Wnt signals can function as positional cues in establishing cell polarity. Dev. Cell.

[42] Jordan S.N., Davies T., Zhuravlev Y., Dumont J., Shirasu-Hiza M., Canman J.C. (2016). Cortical PAR polarity proteins promote robust cytokinesis during asymmetric cell division. J. Cell Biol..

[43] Jamieson C.H.M., Weissman I.L. (2023). Stem-Cell Aging and Pathways to Precancer Evolution. N. Engl. J. Med..

[44] Afify S.M., Seno M. (2019). Conversion of Stem Cells to Cancer Stem Cells: Undercurrent of Cancer Initiation. Cancers.

[45] Abd G.M., Laird M.C., Ku J.C., Li Y. (2023). Hypoxia-induced cancer cell reprogramming: A review on how cancer stem cells arise. Front. Oncol..

[46] Clarke M.F. (2019). Clinical and Therapeutic Implications of Cancer Stem Cells. N. Engl. J. Med..

[47] Najafi M., Farhood B., Mortezaee K. (2019). Cancer stem cells (CSCs) in cancer progression and therapy. J. Cell Physiol..

[48] Aghajani M., Mansoori B., Mohammadi A., Asadzadeh Z., Baradaran B. (2019). New emerging roles of CD133 in cancer stem cell: Signaling pathway and miRNA regulation. J. Cell Physiol..

[49] Barzegar Behrooz A., Syahir A., Ahmad S. (2019). CD133: Beyond a cancer stem cell biomarker. J. Drug Target..

[50] Hassn Mesrati M., Syafruddin S.E., Mohtar M.A., Syahir A. (2021). CD44: A Multifunctional Mediator of Cancer Progression. Biomolecules.

[51] Morath I., Hartmann T.N., Orian-Rousseau V. (2016). CD44: More than a mere stem cell marker. Int. J. Biochem. Cell Biol..

[52] Yang L., Shi P., Zhao G., Xu J., Peng W., Zhang J., Zhang G., Wang X., Dong Z., Chen F. (2020). Targeting cancer stem cell pathways for cancer therapy. Signal Transduct. Target. Ther..

[53] MacLean M.R., Walker O.L., Arun R.P., Fernando W., Marcato P. (2024). Informed by Cancer Stem Cells of Solid Tumors: Advances in Treatments Targeting Tumor-Promoting Factors and Pathways. Int. J. Mol. Sci..

[54] Altevogt P., Sammar M., Huser L., Kristiansen G. (2021). Novel insights into the function of CD24: A driving force in cancer. Int. J. Cancer.

[55] Wang W., Lokman N.A., Barry S.C., Oehler M.K., Ricciardelli C. (2025). LGR5: An emerging therapeutic target for cancer metastasis and chemotherapy resistance. Cancer Metastasis Rev..

[56] Qian W.J., Yan J.S., Gang X.Y., Xu L., Shi S., Li X., Na F.J., Cai L.T., Li H.M., Zhao M.F. (2024). Intercellular adhesion molecule-1 (ICAM-1): From molecular functions to clinical applications in cancer investigation. Biochim. Biophys. Acta Rev. Cancer.

[57] Lavudi K., Nuguri S.M., Pandey P., Kokkanti R.R., Wang Q.E. (2024). ALDH and cancer stem cells: Pathways, challenges, and future directions in targeted therapy. Life Sci..

[58] Kapoor S., Shenoy S.P., Bose B. (2020). CD34 cells in somatic, regenerative and cancer stem cells: Developmental biology, cell therapy, and omics big data perspective. J. Cell Biochem..

[59] Xiong J., Yan L., Zou C., Wang K., Chen M., Xu B., Zhou Z., Zhang D. (2021). Integrins regulate stemness in solid tumor: An emerging therapeutic target. J. Hematol. Oncol..

[60] Meisel C.T., Porcheri C., Mitsiadis T.A. (2020). Cancer Stem Cells, Quo Vadis? The Notch Signaling Pathway in Tumor Initiation and Progression. Cells.

[61] Reya T., Clevers H. (2005). Wnt signalling in stem cells and cancer. Nature.

[62] Holland J.D., Klaus A., Garratt A.N., Birchmeier W. (2013). Wnt signaling in stem and cancer stem cells. Curr. Opin. Cell Biol..

[63] Takebe N., Harris P.J., Warren R.Q., Ivy S.P. (2011). Targeting cancer stem cells by inhibiting Wnt, Notch, and Hedgehog pathways. Nat. Rev. Clin. Oncol..

[64] Vincan E., Barker N. (2008). The upstream components of the Wnt signalling pathway in the dynamic EMT and MET associated with colorectal cancer progression. Clin. Exp. Metastasis.

[65] Takebe N., Nguyen D., Yang S.X. (2014). Targeting notch signaling pathway in cancer: Clinical development advances and challenges. Pharmacol. Ther..

[66] Wu F., Stutzman A., Mo Y.Y. (2007). Notch signaling and its role in breast cancer. Front. Biosci..

[67] Zhou B., Lin W., Long Y., Yang Y., Zhang H., Wu K., Chu Q. (2022). Notch signaling pathway: Architecture, disease, and therapeutics. Signal Transduct. Target. Ther..

[68] Zhao C., Chen A., Jamieson C.H., Fereshteh M., Abrahamsson A., Blum J., Kwon H.Y., Kim J., Chute J.P., Rizzieri D. (2009). Hedgehog signalling is essential for maintenance of cancer stem cells in myeloid leukaemia. Nature.

[69] Feldmann G., Dhara S., Fendrich V., Bedja D., Beaty R., Mullendore M., Karikari C., Alvarez H., Iacobuzio-Donahue C., Jimeno A. (2007). Blockade of hedgehog signaling inhibits pancreatic cancer invasion and metastases: A new paradigm for combination therapy in solid cancers. Cancer Res..

[70] Margadant C., Sonnenberg A. (2010). Integrin-TGF-beta crosstalk in fibrosis, cancer and wound healing. EMBO Rep..

[71] Xu F., Liu C., Zhou D., Zhang L. (2016). TGF-β/SMAD Pathway and Its Regulation in Hepatic Fibrosis. J. Histochem. Cytochem..

[72] Barutcuoglu M., Umur A.S., Vatansever H.S., Umur N., Ozbilgin K., Sayhan S., Selcuki M. (2013). TGF-βs and Smads activities at the site of failed neural tube in the human embryos. Turk. Neurosurg..

[73] Massague J. (2008). TGFbeta in Cancer. Cell.

[74] Saitoh M. (2023). Transcriptional regulation of EMT transcription factors in cancer. Semin. Cancer Biol..

[75] Xue W., Yang L., Chen C., Ashrafizadeh M., Tian Y., Sun R. (2024). Wnt/beta-catenin-driven EMT regulation in human cancers. Cell Mol. Life Sci..

[76] Wang X., Eichhorn P.J.A., Thiery J.P. (2023). TGF-beta, EMT, and resistance to anti-cancer treatment. Semin. Cancer Biol..

[77] Roy S., Sunkara R.R., Parmar M.Y., Shaikh S., Waghmare S.K. (2021). EMT imparts cancer stemness and plasticity: New perspectives and therapeutic potential. Front. Biosci. (Landmark Ed.).

[78] Kanwal R., Esposito J.E., Jawed B., Zakir S.K., Pulcini R., Martinotti R., Botteghi M., Gaudio F., Martinotti S., Toniato E. (2025). Exploring the Role of Epithelial-Mesenchymal Transcriptional Factors Involved in Hematological Malignancy and Solid Tumors: A Systematic Review. Cancers.

[79] Khan A.Q., Hasan A., Mir S.S., Rashid K., Uddin S., Steinhoff M. (2024). Exploiting transcription factors to target EMT and cancer stem cells for tumor modulation and therapy. Semin. Cancer Biol..

[80] Velychko S., Adachi K., Kim K.P., Hou Y., MacCarthy C.M., Wu G., Schöler H.R. (2019). Excluding Oct4 from Yamanaka Cocktail Unleashes the Developmental Potential of iPSCs. Cell Stem Cell.

[81] Baillie R., Itinteang T., Yu H.H., Brasch H.D., Davis P.F., Tan S.T. (2016). Cancer stem cells in moderately differentiated oral tongue squamous cell carcinoma. J. Clin. Pathol..

[82] Chai S., Ng K.Y., Tong M., Lau E.Y., Lee T.K., Chan K.W., Yuan Y.F., Cheung T.T., Cheung S.T., Wang X.Q. (2016). Octamer 4/microRNA-1246 signaling axis drives Wnt/beta-catenin activation in liver cancer stem cells. Hepatology.

[83] Liu C., Peng G., Jing N. (2018). TGF-β signaling pathway in early mouse development and embryonic stem cells. Acta Biochim. Biophys. Sin..

[84] Yin X., Zhang B.H., Zheng S.S., Gao D.M., Qiu S.J., Wu W.Z., Ren Z.G. (2015). Coexpression of gene Oct4 and Nanog initiates stem cell characteristics in hepatocellular carcinoma and promotes epithelial-mesenchymal transition through activation of Stat3/Snail signaling. J. Hematol. Oncol..

[85] Li S.W., Wu X.L., Dong C.L., Xie X.Y., Wu J.F., Zhang X. (2015). The differential expression of OCT4 isoforms in cervical carcinoma. PLoS ONE.

[86] Lu Y., Zhu H., Shan H., Lu J., Chang X., Li X., Lu J., Fan X., Zhu S., Wang Y. (2013). Knockdown of Oct4 and Nanog expression inhibits the stemness of pancreatic cancer cells. Cancer Lett..

[87] Novak D., Huser L., Elton J.J., Umansky V., Altevogt P., Utikal J. (2020). SOX2 in development and cancer biology. Semin. Cancer Biol..

[88] Leis O., Eguiara A., Lopez-Arribillaga E., Alberdi M.J., Hernandez-Garcia S., Elorriaga K., Pandiella A., Rezola R., Martin A.G. (2012). Sox2 expression in breast tumours and activation in breast cancer stem cells. Oncogene.

[89] Piva M., Domenici G., Iriondo O., Rabano M., Simoes B.M., Comaills V., Barredo I., Lopez-Ruiz J.A., Zabalza I., Kypta R. (2014). Sox2 promotes tamoxifen resistance in breast cancer cells. EMBO Mol. Med..

[90] Boumahdi S., Driessens G., Lapouge G., Rorive S., Nassar D., Le Mercier M., Delatte B., Caauwe A., Lenglez S., Nkusi E. (2014). SOX2 controls tumour initiation and cancer stem-cell functions in squamous-cell carcinoma. Nature.

[91] Bass A.J., Watanabe H., Mermel C.H., Yu S., Perner S., Verhaak R.G., Kim S.Y., Wardwell L., Tamayo P., Gat-Viks I. (2009). SOX2 is an amplified lineage-survival oncogene in lung and esophageal squamous cell carcinomas. Nat. Genet..

[92] Berezovsky A.D., Poisson L.M., Cherba D., Webb C.P., Transou A.D., Lemke N.W., Hong X., Hasselbach L.A., Irtenkauf S.M., Mikkelsen T. (2014). Sox2 promotes malignancy in glioblastoma by regulating plasticity and astrocytic differentiation. Neoplasia.

[93] Ferone G., Song J.Y., Sutherland K.D., Bhaskaran R., Monkhorst K., Lambooij J.P., Proost N., Gargiulo G., Berns A. (2016). SOX2 Is the Determining Oncogenic Switch in Promoting Lung Squamous Cell Carcinoma from Different Cells of Origin. Cancer Cell.

[94] Zinin N., Adameyko I., Wilhelm M., Fritz N., Uhlén P., Ernfors P., Henriksson M.A. (2014). MYC proteins promote neuronal differentiation by controlling the mode of progenitor cell division. EMBO Rep..

[95] Dong Y., Tu R., Liu H., Qing G. (2020). Regulation of cancer cell metabolism: Oncogenic MYC in the driver’s seat. Signal Transduct. Target. Ther..

[96] Dang C.V. (2012). MYC on the path to cancer. Cell.

[97] Dupont S., Morsut L., Aragona M., Enzo E., Giulitti S., Cordenonsi M., Zanconato F., Le Digabel J., Forcato M., Bicciato S. (2011). Role of YAP/TAZ in mechanotransduction. Nature.

[98] Barry E.R., Camargo F.D. (2013). The Hippo superhighway: Signaling crossroads converging on the Hippo/Yap pathway in stem cells and development. Curr. Opin. Cell Biol..

[99] Tschaharganeh D.F., Chen X., Latzko P., Malz M., Gaida M.M., Felix K., Ladu S., Singer S., Pinna F., Gretz N. (2013). Yes-associated protein up-regulates Jagged-1 and activates the Notch pathway in human hepatocellular carcinoma. Gastroenterology.

[100] Ganguly K., Krishn S.R., Rachagani S., Jahan R., Shah A., Nallasamy P., Rauth S., Atri P., Cox J.L., Pothuraju R. (2021). Secretory Mucin 5AC Promotes Neoplastic Progression by Augmenting KLF4-Mediated Pancreatic Cancer Cell Stemness. Cancer Res..

[101] Kress T.R., Sabo A., Amati B. (2015). MYC: Connecting selective transcriptional control to global RNA production. Nat. Rev. Cancer.

[102] Qi X.T., Li Y.L., Zhang Y.Q., Xu T., Lu B., Fang L., Gao J.Q., Yu L.S., Zhu D.F., Yang B. (2019). KLF4 functions as an oncogene in promoting cancer stem cell-like characteristics in osteosarcoma cells. Acta Pharmacol. Sin..

[103] Okuda H., Xing F., Pandey P.R., Sharma S., Watabe M., Pai S.K., Mo Y.Y., Iiizumi-Gairani M., Hirota S., Liu Y. (2013). miR-7 suppresses brain metastasis of breast cancer stem-like cells by modulating KLF4. Cancer Res..

[104] Zou H., Chen H., Zhou Z., Wan Y., Liu Z. (2019). ATXN3 promotes breast cancer metastasis by deubiquitinating KLF4. Cancer Lett..

[105] Huang W., Zhong Z., Luo C., Xiao Y., Li L., Zhang X., Yang L., Xiao K., Ning Y., Chen L. (2019). The miR-26a/AP-2alpha/Nanog signaling axis mediates stem cell self-renewal and temozolomide resistance in glioma. Theranostics.

[106] Sun B., Xu L., Bi W., Ou W.B. (2022). SALL4 Oncogenic Function in Cancers: Mechanisms and Therapeutic Relevance. Int. J. Mol. Sci..

[107] Zhang L., Xu Z., Xu X., Zhang B., Wu H., Wang M., Zhang X., Yang T., Cai J., Yan Y. (2014). SALL4, a novel marker for human gastric carcinogenesis and metastasis. Oncogene.

[108] Sher G., Masoodi T., Patil K., Akhtar S., Kuttikrishnan S., Ahmad A., Uddin S. (2022). Dysregulated FOXM1 signaling in the regulation of cancer stem cells. Semin. Cancer Biol..

[109] Wang W., Liu W., Chen Q., Yuan Y., Wang P. (2022). Targeting CSC-related transcription factors by E3 ubiquitin ligases for cancer therapy. Semin. Cancer Biol..

[110] Guo Y., Liu S., Wang P., Zhao S., Wang F., Bing L., Zhang Y., Ling E.A., Gao J., Hao A. (2011). Expression profile of embryonic stem cell-associated genes Oct4, Sox2 and Nanog in human gliomas. Histopathology.

[111] Jen J., Tang Y.A., Lu Y.H., Lin C.C., Lai W.W., Wang Y.C. (2017). Oct4 transcriptionally regulates the expression of long non-coding RNAs NEAT1 and MALAT1 to promote lung cancer progression. Mol. Cancer.

[112] Wen J., Park J.Y., Park K.H., Chung H.W., Bang S., Park S.W., Song S.Y. (2010). Oct4 and Nanog expression is associated with early stages of pancreatic carcinogenesis. Pancreas.

[113] Guo J., Grow E.J., Yi C., Mlcochova H., Maher G.J., Lindskog C., Murphy P.J., Wike C.L., Carrell D.T., Goriely A. (2017). Chromatin and Single-Cell RNA-Seq Profiling Reveal Dynamic Signaling and Metabolic Transitions during Human Spermatogonial Stem Cell Development. Cell Stem Cell.

[114] He Z., Jiang J., Kokkinaki M., Dym M. (2009). Nodal signaling via an autocrine pathway promotes proliferation of mouse spermatogonial stem/progenitor cells through Smad2/3 and Oct-4 activation. Stem Cells.

[115] Chen W., Wang Y.J. (2025). Multifaceted roles of OCT4 in tumor microenvironment: Biology and therapeutic implications. Oncogene.

[116] Gordeeva O. (2019). TGFbeta Family Signaling Pathways in Pluripotent and Teratocarcinoma Stem Cells’ Fate Decisions: Balancing Between Self-Renewal, Differentiation, and Cancer. Cells.

[117] Wang Y., Frederick J., Medina K.I., Bartom E.T., Almassalha L.M., Zhang Y., Wodarcyk G., Huang H., Ye I.C., Gong R. (2025). Chromatin Organization Governs Transcriptional Response and Plasticity of Cancer Stem Cells. Adv. Sci..

[118] Vishnubalaji R., Shaath H., Al-Alwan M., Abdelalim E.M., Alajez N.M. (2022). Reciprocal interplays between MicroRNAs and pluripotency transcription factors in dictating stemness features in human cancers. Semin. Cancer Biol..

[119] Rodriguez-Brenes I.A., Komarova N.L., Wodarz D. (2011). Evolutionary dynamics of feedback escape and the development of stem-cell-driven cancers. Proc. Natl. Acad. Sci. USA.

[120] Weiss L.D., van den Driessche P., Lowengrub J.S., Wodarz D., Komarova N.L. (2021). Effect of feedback regulation on stem cell fractions in tissues and tumors: Understanding chemoresistance in cancer. J. Theor. Biol..

[121] Esteller M. (2008). Epigenetics in cancer. N. Engl. J. Med..

[122] Sharma S., Kelly T.K., Jones P.A. (2010). Epigenetics in cancer. Carcinogenesis.

[123] Wang Q., Liang N., Yang T., Li Y., Li J., Huang Q., Wu C., Sun L., Zhou X., Cheng X. (2021). DNMT1-mediated methylation of BEX1 regulates stemness and tumorigenicity in liver cancer. J. Hepatol..

[124] Zhang G., Lu J., Yang M., Wang Y., Liu H., Xu C. (2020). Elevated GALNT10 expression identifies immunosuppressive microenvironment and dismal prognosis of patients with high grade serous ovarian cancer. Cancer Immunol. Immunother..

[125] Zhao F.Y., Zhang Q., Wang J.M., Jiang J.Y., Huyan L.Y., Liu B.Q., Yan J., Li C., Wang H.Q. (2021). BAG3 epigenetically regulates GALNT10 expression via WDR5 and facilitates the stem cell-like properties of platin-resistant ovarian cancer cells. Biochim. Biophys. Acta Mol. Cell Res..

[126] Abu Sailik F., Emerald B.S., Ansari S.A. (2024). Opening and changing: Mammalian SWI/SNF complexes in organ development and carcinogenesis. Open Biol..

[127] Bracken A.P., Brien G.L., Verrijzer C.P. (2019). Dangerous liaisons: Interplay between SWI/SNF, NuRD, and Polycomb in chromatin regulation and cancer. Genes. Dev..

[128] Wang L., Tang J. (2023). SWI/SNF complexes and cancers. Gene.

[129] Kadoch C., Copeland R.A., Keilhack H. (2016). PRC2 and SWI/SNF Chromatin Remodeling Complexes in Health and Disease. Biochemistry.

[130] Galassi C., Manic G., Esteller M., Galluzzi L., Vitale I. (2025). Epigenetic regulation of cancer stemness. Signal Transduct. Target. Ther..

[131] Calin G.A., Croce C.M. (2006). MicroRNA signatures in human cancers. Nat. Rev. Cancer.

[132] Medina P.P., Nolde M., Slack F.J. (2010). OncomiR addiction in an in vivo model of microRNA-21-induced pre-B-cell lymphoma. Nature.

[133] Valadi H., Ekstrom K., Bossios A., Sjostrand M., Lee J.J., Lotvall J.O. (2007). Exosome-mediated transfer of mRNAs and microRNAs is a novel mechanism of genetic exchange between cells. Nat. Cell Biol..

[134] Shang A., Wang X., Gu C., Liu W., Sun J., Zeng B., Chen C., Ji P., Wu J., Quan W. (2020). Exosomal miR-183-5p promotes angiogenesis in colorectal cancer by regulation of FOXO1. Aging.

[135] Hoshino A., Costa-Silva B., Shen T.L., Rodrigues G., Hashimoto A., Tesic Mark M., Molina H., Kohsaka S., Di Giannatale A., Ceder S. (2015). Tumour exosome integrins determine organotropic metastasis. Nature.

[136] Chen J., Xu Z., Huang H., Tang Y., Shan H., Xiao F. (2023). SETD1A drives stemness by reprogramming the epigenetic landscape in hepatocellular carcinoma stem cells. JCI Insight.

[137] Fisher M.L., Balinth S., Hwangbo Y., Wu C., Ballon C., Wilkinson J.E., Goldberg G.L., Mills A.A. (2021). BRD4 Regulates Transcription Factor DeltaNp63alpha to Drive a Cancer Stem Cell Phenotype in Squamous Cell Carcinomas. Cancer Res..

[138] O’Brien K., Lowry M.C., Corcoran C., Martinez V.G., Daly M., Rani S., Gallagher W.M., Radomski M.W., MacLeod R.A., O’Driscoll L. (2015). miR-134 in extracellular vesicles reduces triple-negative breast cancer aggression and increases drug sensitivity. Oncotarget.

[139] Aramini B., Masciale V., Grisendi G., Bertolini F., Maur M., Guaitoli G., Chrystel I., Morandi U., Stella F., Dominici M. (2022). Dissecting Tumor Growth: The Role of Cancer Stem Cells in Drug Resistance and Recurrence. Cancers.

[140] Barbato L., Bocchetti M., Di Biase A., Regad T. (2019). Cancer Stem Cells and Targeting Strategies. Cells.

[141] Mayani H., Chavez-Gonzalez A., Vazquez-Santillan K., Contreras J., Guzman M.L. (2022). Cancer Stem Cells: Biology and Therapeutic Implications. Arch. Med. Res..

[142] Mimeault M., Batra S.K. (2014). Altered gene products involved in the malignant reprogramming of cancer stem/progenitor cells and multitargeted therapies. Mol. Aspects Med..

[143] Paul R., Dorsey J.F., Fan Y. (2022). Cell plasticity, senescence, and quiescence in cancer stem cells: Biological and therapeutic implications. Pharmacol. Ther..

[144] Huang T., Song X., Xu D., Tiek D., Goenka A., Wu B., Sastry N., Hu B., Cheng S.Y. (2020). Stem cell programs in cancer initiation, progression, and therapy resistance. Theranostics.

[145] Dzobo K., Senthebane D.A., Ganz C., Thomford N.E., Wonkam A., Dandara C. (2020). Advances in Therapeutic Targeting of Cancer Stem Cells within the Tumor Microenvironment: An Updated Review. Cells.

[146] Esmaeili S.A., Sahranavard S., Salehi A., Bagheri V. (2021). Selectively targeting cancer stem cells: Current and novel therapeutic strategies and approaches in the effective eradication of cancer. IUBMB Life.

[147] Angelova E., Audette C., Kovtun Y., Daver N., Wang S.A., Pierce S., Konoplev S.N., Khogeer H., Jorgensen J.L., Konopleva M. (2019). CD123 expression patterns and selective targeting with a CD123-targeted antibody-drug conjugate (IMGN632) in acute lymphoblastic leukemia. Haematologica.

[148] Daver N.G., Montesinos P., DeAngelo D.J., Wang E.S., Papadantonakis N., Todisco E., Sweet K.L., Pemmaraju N., Lane A.A., Torres-Minana L. (2024). Pivekimab sunirine (IMGN632), a novel CD123-targeting antibody-drug conjugate, in relapsed or refractory acute myeloid leukaemia: A phase 1/2 study. Lancet Oncol..

[149] Takebe N., Miele L., Harris P.J., Jeong W., Bando H., Kahn M., Yang S.X., Ivy S.P. (2015). Targeting Notch, Hedgehog, and Wnt pathways in cancer stem cells: Clinical update. Nat. Rev. Clin. Oncol..

[150] Schmults C.D., Blitzblau R., Aasi S.Z., Alam M., Amini A., Bibee K., Bordeaux J., Chen P.L., Contreras C.M., DiMaio D. (2023). Basal Cell Skin Cancer, Version 2.2024, NCCN Clinical Practice Guidelines in Oncology. J. Natl. Compr. Cancer Netw..

[151] Li C., Heidt D.G., Dalerba P., Burant C.F., Zhang L., Adsay V., Wicha M., Clarke M.F., Simeone D.M. (2007). Identification of pancreatic cancer stem cells. Cancer Res..

[152] Lee C.J., Dosch J., Simeone D.M. (2008). Pancreatic cancer stem cells. J. Clin. Oncol..

[153] Mallya K., Gautam S.K., Aithal A., Batra S.K., Jain M. (2021). Modeling pancreatic cancer in mice for experimental therapeutics. Biochim. Biophys. Acta Rev. Cancer.

[154] Stoica A.F., Chang C.H., Pauklin S. (2020). Molecular Therapeutics of Pancreatic Ductal Adenocarcinoma: Targeted Pathways and the Role of Cancer Stem Cells. Trends Pharmacol. Sci..

[155] Khan M.A., Srivastava S.K., Zubair H., Patel G.K., Arora S., Khushman M., Carter J.E., Gorman G.S., Singh S., Singh A.P. (2020). Co-targeting of CXCR4 and hedgehog pathways disrupts tumor-stromal crosstalk and improves chemotherapeutic efficacy in pancreatic cancer. J. Biol. Chem..

[156] Feldmann G., Habbe N., Dhara S., Bisht S., Alvarez H., Fendrich V., Beaty R., Mullendore M., Karikari C., Bardeesy N. (2008). Hedgehog inhibition prolongs survival in a genetically engineered mouse model of pancreatic cancer. Gut.

[157] De Jesus-Acosta A., Sugar E.A., O’Dwyer P.J., Ramanathan R.K., Von Hoff D.D., Rasheed Z., Zheng L., Begum A., Anders R., Maitra A. (2020). Phase 2 study of vismodegib, a hedgehog inhibitor, combined with gemcitabine and nab-paclitaxel in patients with untreated metastatic pancreatic adenocarcinoma. Br. J. Cancer.

[158] Kim E.J., Sahai V., Abel E.V., Griffith K.A., Greenson J.K., Takebe N., Khan G.N., Blau J.L., Craig R., Balis U.G. (2014). Pilot clinical trial of hedgehog pathway inhibitor GDC-0449 (vismodegib) in combination with gemcitabine in patients with metastatic pancreatic adenocarcinoma. Clin. Cancer Res..

[159] Melisi D., Oh D.Y., Hollebecque A., Calvo E., Varghese A., Borazanci E., Macarulla T., Merz V., Zecchetto C., Zhao Y. (2021). Safety and activity of the TGFbeta receptor I kinase inhibitor galunisertib plus the anti-PD-L1 antibody durvalumab in metastatic pancreatic cancer. J. Immunother. Cancer.

[160] Melisi D., Garcia-Carbonero R., Macarulla T., Pezet D., Deplanque G., Fuchs M., Trojan J., Oettle H., Kozloff M., Cleverly A. (2018). Galunisertib plus gemcitabine vs. gemcitabine for first-line treatment of patients with unresectable pancreatic cancer. Br. J. Cancer.

[161] Singh S.K., Hawkins C., Clarke I.D., Squire J.A., Bayani J., Hide T., Henkelman R.M., Cusimano M.D., Dirks P.B. (2004). Identification of human brain tumour initiating cells. Nature.

[162] Chu Q., Orr B.A., Semenkow S., Bar E.E., Eberhart C.G. (2013). Prolonged inhibition of glioblastoma xenograft initiation and clonogenic growth following in vivo Notch blockade. Clin. Cancer Res..

[163] Fan X., Khaki L., Zhu T.S., Soules M.E., Talsma C.E., Gul N., Koh C., Zhang J., Li Y.M., Maciaczyk J. (2010). NOTCH pathway blockade depletes CD133-positive glioblastoma cells and inhibits growth of tumor neurospheres and xenografts. Stem Cells.

[164] Wang S., Gu S., Chen J., Yuan Z., Liang P., Cui H. (2024). Mechanism of Notch Signaling Pathway in Malignant Progression of Glioblastoma and Targeted Therapy. Biomolecules.

[165] Agosti E., Antonietti S., Ius T., Fontanella M.M., Zeppieri M., Panciani P.P. (2024). Glioma Stem Cells as Promoter of Glioma Progression: A Systematic Review of Molecular Pathways and Targeted Therapies. Int. J. Mol. Sci..

[166] Peereboom D.M., Ye X., Mikkelsen T., Lesser G.J., Lieberman F.S., Robins H.I., Ahluwalia M.S., Sloan A.E., Grossman S.A. (2021). A Phase II and Pharmacodynamic Trial of RO4929097 for Patients with Recurrent/Progressive Glioblastoma. Neurosurgery.

[167] Xu R., Shimizu F., Hovinga K., Beal K., Karimi S., Droms L., Peck K.K., Gutin P., Iorgulescu J.B., Kaley T. (2016). Molecular and Clinical Effects of Notch Inhibition in Glioma Patients: A Phase 0/I Trial. Clin. Cancer Res..

[168] Tanaka S., Nakada M., Yamada D., Nakano I., Todo T., Ino Y., Hoshii T., Tadokoro Y., Ohta K., Ali M.A. (2015). Strong therapeutic potential of gamma-secretase inhibitor MRK003 for CD44-high and CD133-low glioblastoma initiating cells. J. Neuro-Oncology.

[169] Gutova M., Hibbard J.C., Ma E., Natri H.M., Adhikarla V., Chimge N.O., Qiu R., Nguyen C., Melendez E., Aguilar B. (2024). Targeting Wnt signaling for improved glioma immunotherapy. Front. Immunol..

[170] Behrooz A.B., Syahir A. (2021). Could We Address the Interplay Between CD133, Wnt/beta-Catenin, and TERT Signaling Pathways as a Potential Target for Glioblastoma Therapy?. Front. Oncol..

[171] Bos E.M., Binda E., Verploegh I.S.C., Wembacher E., Hoefnagel D., Balvers R.K., Korporaal A.L., Conidi A., Warnert E.A.H., Trivieri N. (2023). Local delivery of hrBMP4 as an anticancer therapy in patients with recurrent glioblastoma: A first-in-human phase 1 dose escalation trial. Mol. Cancer.

[172] Zhao X., Sun Q., Dou C., Chen Q., Liu B. (2019). BMP4 inhibits glioblastoma invasion by promoting E-cadherin and claudin expression. Front. Biosci. (Landmark Ed.).

[173] Verploegh I.S.C., Conidi A., El Hassnaoui H., Verhoeven F.A.M., Korporaal A.L., Ntafoulis I., van den Hout M., Brouwer R.W.W., Lamfers M.L.M., van I.W.F.J. (2024). BMP4 and Temozolomide Synergize in the Majority of Patient-Derived Glioblastoma Cultures. Int. J. Mol. Sci..

[174] Gulati G.S., D’Silva J.P., Liu Y., Wang L., Newman A.M. (2025). Profiling cell identity and tissue architecture with single-cell and spatial transcriptomics. Nat. Rev. Mol. Cell Biol..

[175] Schork N.J. (2015). Personalized medicine: Time for one-person trials. Nature.

